# Diffusion‐weighted MRI of the lung at 3T evaluated using echo‐planar‐based and single‐shot turbo spin‐echo‐based acquisition techniques for radiotherapy applications

**DOI:** 10.1002/acm2.12493

**Published:** 2018-11-12

**Authors:** Neelam Tyagi, Michelle Cloutier, Kristen Zakian, Joseph O. Deasy, Margie Hunt, Andreas Rimner

**Affiliations:** ^1^ Department of Medical Physics Memorial Sloan Kettering Cancer Center New York NY USA; ^2^ Department of Radiation Oncology Memorial Sloan Kettering Cancer Center New York NY USA

**Keywords:** DW‐MRI, echo‐planar imaging, non‐small cell lung cancer, turbo spin‐echo

## Abstract

**Purpose:**

To compare single‐shot echo‐planar (SS‐EPI)‐based and turbo spin‐echo (SS‐TSE)‐based diffusion‐weighted imaging (DWI) in Non‐Small Cell Lung Cancer (NSCLC) patients and to characterize the distributions of apparent diffusion coefficient (ADC) values generated by the two techniques.

**Methods:**

Ten NSCLC patients were enrolled in a prospective IRB‐approved study to compare and optimize DWI using EPI and TSE‐based techniques for radiotherapy planning. The imaging protocol included axial T2w, EPI‐based DWI and TSE‐based DWI on a 3 T Philips scanner. Both EPI‐based and TSE‐based DWI sequences used three b values (0, 400, and 800 s/mm^2^). The acquisition times for EPI‐based and TSE‐based DWI were 5 and 8 min, respectively. DW‐MR images were manually coregistered with axial T2w images, and tumor volume contoured on T2w images were mapped onto the DWI scans. A pixel‐by‐pixel fit of tumor ADC was calculated based on monoexponential signal behavior. Tumor ADC mean, standard deviation, kurtosis, and skewness were calculated and compared between EPI and TSE‐based DWI. Image distortion and ADC values between the two techniques were also quantified using fieldmap analysis and a NIST traceable ice‐water diffusion phantom, respectively.

**Results:**

The mean ADC for EPI and TSE‐based DWI were 1.282 ± 0.42 × 10^−3^ and 1.211 ± 0.31 × 10^−3^ mm^2^/s. The average skewness and kurtosis were 0.14 ± 0.4 and 2.43 ± 0.40 for DWI‐EPI and −0.06 ± 0.69 and 2.89 ± 0.62 for DWI‐TSE. Fieldmap analysis showed a mean distortion of 13.72 ± 8.12 mm for GTV for DWI‐EPI and 0.61 ± 0.4 mm for DWI‐TSE. ADC values obtained using the diffusion phantom for the two techniques were within 0.03 × 10^−3^ mm^2^/s with respect to each other as well as the established values.

**Conclusions:**

Diffusion‐weighted turbo spin‐echo shows better geometrical accuracy compared to DWI‐EPI. Mean ADC values were similar with both acquisitions but the shape of the histograms was different based on the skewness and kurtosis values. The impact of differences in respiratory technique on ADC values requires further investigation.

## 1 INTRODUCTION

Magnetic resonance imaging (MRI) in the lung is challenging due to breathing and cardiac motion. At the same time, MRI in the lung is also appealing because any pathology in the lung will have a higher proton density than surrounding normal tissue and therefore a higher MR signal with a strong inherent contrast against the dark background. Recently, there has been tremendous interest in the use of diffusion‐weighted MRI in lung cancer for diagnosis, staging and response assessment.^1^, ^2^, ^3^, ^4^, ^5^, ^6^, ^7^ Diffusion‐weighted imaging (DWI) is typically acquired using an echo‐planar imaging (EPI)‐based acquisition that provides high signal‐to‐noise ratio (SNR) and is very fast to minimize the effects of physiological motions arising from respiration, cardiac or any bulk motion.^8^

In an EPI acquisition, the echo trains are formed by gradient pulses, which do not rephase spins that have become dephased due to intravoxel field inhomogeneity. Therefore, the EPI signal can be greatly reduced in the presence of large differences in magnetic susceptibility at air/tissue interfaces due to rapid intravoxel dephasing and the extremely short resultant T2*.^9^ In addition to signal loss, field inhomogeneity results in image distortion when spins encoded by frequency are mapped to the incorrect location. The spatial shift is proportional to the ratio of the field inhomogeneity over the voxel (in Hz) to the voxel acquisition bandwidth (BW) and can be several mm or more in EPI where voxel bandwidths are low. Furthermore, the effect of susceptibility differences scales with field strength and is therefore more severe on 3 T MR scanners. On the other hand, if the echo train is formed by radiofrequency pulses, such as turbo spin‐echo (TSE)/fast spin‐echo (FSE) based acquisition,^10^ the effect of static field inhomogeneities will be refocused, increasing the signal at a given echo time and permitting longer sampling windows, higher voxel bandwidths and less spatial distortion than EPI‐based DWI. The extent of distortion in DWI scans may have an impact on tumor characterization, tumor delineation and response assessment. Although numerous studies have utilized EPI‐based acquisition for the lung, only one study has used an FSE‐based DWI technique.^2^ Matoba, et al. showed the use of split acquisition of fast spin‐echo signals, or SPLICE,^11^ for diffusion imaging in the lung. Currently, there are no studies directly comparing the use of TSE‐ and EPI‐based DWI in the lung. The goal of this study was to characterize geometric accuracy and apparent diffusion coefficient (ADC) histograms derived from single‐shot EPI‐ and TSE‐based DWI acquisitions.

## 2 MATERIALS AND METHODS

### 2.A DWI‐TSE sequence implementation

In DWI‐TSE, diffusion gradients are applied before and after the 180‐degree refocusing pulse to allow for diffusion acquisition using a TSE sequence. The single‐shot TSE‐diffusion pulse sequence provided by Philips healthcare incorporates the following features in order to shorten echo train length, and minimize blurring: (a) averaging of modulus data instead of complex data to minimize the effect of phase differences between echoes, (b) a short refocusing pulse that has a less sharp profile than the standard refocusing pulse but reduces echo spacing, and (c) sensitivity encoding‐based parallel imaging.^12^

### 2.B Phantom study

A National Institute of Standards and Technology traceable, temperature‐controlled ice‐water diffusion phantom (High Precision Devices, Inc, Boulder, CO, USA) was scanned using both single‐shot echo‐planar (SS‐EPI)‐based and turbo spin‐echo (SS‐TSE)‐based DWI‐based acquisitions at 0°C.^13^ The phantom was scanned using a 16‐element head coil on the 3 T Philips Ingenia scanner with four different b values: 0, 500, 900, and 2000, with the established scan parameter values.^13^ The phantom consisted of 13 vials containing 30 ml of polymer polyvinylpyrrolidone in aqueous solution at various concentrations. The phantom scan was repeated twice 2 month apart, and two sets of ADC measurements were performed for both EPI and TSE acquisition on each day. A 1 cm diameter region of interest (ROI) was defined in the center of each vial to calculate the mean ADC and standard deviation for each technique.

### 2.C Patient selection and Imaging protocol

Ten patients (eight men, two women; median age: 64 yr (range 51–74 yr) with locally advanced Non‐Small Cell Lung Cancer undergoing chemoradiation were enrolled in a prospective IRB‐approved study to undergo DWI using both the SS‐EPI‐ and SS‐TSE‐based techniques. Table 1 shows the patient characteristics, such as age, diagnosis, TNM status,^14^ tumor histology, and the tumor volume, as measured on T2‐weighted (T2w) MRI.

**Table 1:** Non‐small cell lung cancer (NSCLC) Patient characteristics. The TNM stage system developed by the American Joint Commission on Cancer (AJCC) was used, where T value describes size and extent of primary tumor, N indicates lymph node extension, and M indicates distant metastatic status.^14^

Pat	Age	Diagnosis	TNM Status	Histologic tumor type	T2 tumor volume (CC)
1	87	NSCLC	T2N0	Squamous carcinoma	88.2
2	60	NSCLC	T2aN0M0	Adenocarcinoma	45.4
3	70	NSCLC	T1N3M0	Adenocarcinoma	5.4
4	51	NSCLC	T3‐4N0M0	Sarcomatoid carcinoma	258.6
5	74	NSCLC	T2aN3M0	Adenocarcinoma	118
6	62	NSCLC	T2N2M0	Adenocarcinoma	90.2
7	60	NSCLC	T2N3M0	Adenocarcinoma	132.8
8	71	NSCLC	T2N3M1	Adenocarcinoma	59.1
9	65	NSCLC	T4N2M0	Squamous carcinoma	211.4
10	65	NSCLC	T4N1M0	Adenocarcinoma	136.2

The imaging protocol included anatomical high‐resolution axial T2w, EPI‐based DWI‐ and TSE‐based DW‐MRI using a 3 T Philips Ingenia scanner (Philips Medical Systems). All the patients were scanned using a 16‐element phased array anterior coil and a 44‐element posterior coil. Sequence parameters were determined to optimize image quality within clinically acceptable scan times. EPI‐based DWI was performed using a single‐shot respiratory‐triggered echo‐planar imaging sequence with three b values (0, 400, and 800 s/mm^2^) and single diffusion gradient direction (AP× RL × FH = 300 × 300 × 97, Acquisition voxel size = 2.5 × 2.5 mm^2^, TR = 4000–6000 ms, TE = 49 ms, flip angle = 90°, 120 × 120, slice thickness: 6 mm, gap = 1 mm, number of slices = 14, number of signal averages (NSA) = 3 and BW = 34.4 Hz/pixel). TSE‐based DWI was performed using a free breathing, single‐shot TSE‐based image sequence (AP × RL × FH = 230 × 195 × 97, Acquisition voxel = 1.8 × 1.7 mm^2^, TR = 8500, TE = 68, FA = 90, 128 × 115, gap = 1 mm, NSA = 6, BW = 757 Hz/pixel) using the same three b values. After reconstruction, the effective voxels for both acquisitions were 1.8 × 1.8 × 6 mm^3^. Table 2 compares the EPI vs TSE parameters in detail. Anatomical scans included a two‐dimensional T2‐w FSE sequence (TR/TE = 3000–6000/120 ms, slice thickness = 2.5 mm and in‐plane resolution of 1.1 × 0.97 mm^2^, FA = 90°, number of slices = 43, NSA = 2). A dual‐echo (TE1/TE2/TR = 2.3/4.626/30 ms, 3.5 × 3.5 × 4 mm3, FA = 60°) three‐dimensional gradient echo sequence matching the anatomical positioning was also acquired to obtain B0 field maps on three representative cases.

**Table 2:** Single‐shot echo‐planar (EPI) and single‐shot turbo spin‐echo (TSE) diffusion‐weighted imaging (DWI) sequence parameters that were chosen for optimal image quality within clinically acceptable scan times

	DWI‐EPI	DWI‐TSE
TR	4000 – 6000 ms	8500 – 9000 ms
TE	49 ms	68 ms
Matrix	120 × 120	128 × 115
Slice thickness	6 mm	6 mm
Field of view	300 × 300 × 97 mm^3^	230 × 195 × 97 mm^3^
Pixel bandwidth (Hz)	34.4	757
Number of signal averages (NSA)	4	6
b values (s/mm^2^)	0, 400, 800	0, 400, 800
Sensitivity encoding acceleration factor	4	2
Fat suppression technique	SPIR	SPIR
Echo train length (ETL) in ms	41	106
Trigger type	Respiratory navigator triggered	Free breathing
Phase encoding direction	Left‐to‐right	Left‐to‐right
Acquisition time	3‐5 min	8 min

### 2.D DWI analysis

**Figure 1:**
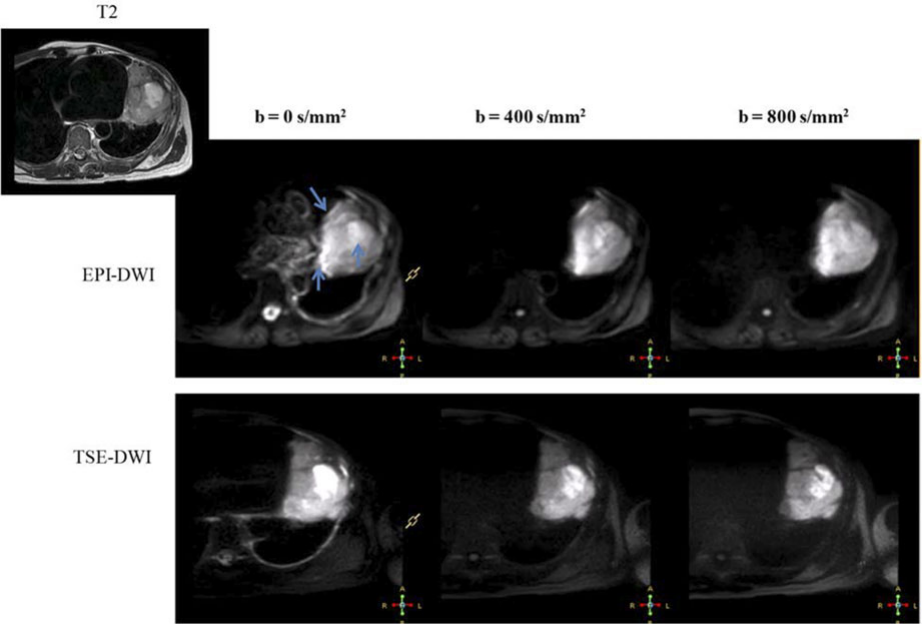
Diffusion‐weighted echo‐planar imaging (EPI‐DWI) and diffusion‐weighted turbo spin‐echo (TSE‐DWI) images of an example patient (patient 10). Images corresponding to b = 0, 400, and 800 s/mm^2^ are shown. Top left image shows the T2w magnetic resonance image of the tumor location. Blue arrows show the regions of susceptibility artifacts of EPI‐DWI.

Diffusion signal decay was modeled exponentially as a function of b value where b is a factor representing diffusion weighting. Quantification of this signal loss is performed by calculating the ADC from:

(1) $$S\left(b\right)=S\left(0\right)e^{-b*ADC}$$

where S(b) is signal intensity measured for a given b value, and S(0) is the signal intensity for b = 0 s/mm^2^. Anatomical T2‐w images and b = 0 DW images were imported into MIM VISTA^TM^ for contouring EPI‐DWI and TSE‐DWI tumor volumes. Tumor contours drawn on the anatomic T2‐w images were transferred to the DWI images after manual registration. Figure 1 shows EPI‐ and TSE‐based DWI and T2w MRI of an example case. In the EPI images, susceptibility‐related signal “pile up” can be seen at the tumor edge (arrows).

### 2.E Distortion evaluation

To evaluate the extent of patient‐specific distortions in lung DWI images, B_0_ maps (in Hz), were derived from two gradient echo images with different echo times and obtained for three cases. The change in MR signal phase from one echo to the next is proportional to both the field inhomogeneity in that voxel and the echo time difference.^15^ B_0_ maps were converted to pixel shift maps based on the BW, as shown by the equations below.

$$\Delta B_{0}=\frac{\phi_{2}-\phi_{1}}{\gamma (T_{E,2}-T_{E,1})}$$

$$\Delta x=\frac{\Delta B_{0}\left(x,y\right)}{BW_{x}}\Delta v_{x}$$

where Δ*B*_0_ is the *B*0 field distortion in Hz, *φ*_1_ and *φ*_2_ are the phase values of two images, TE_1_ and TE_2_ are the echo times of the two images, *γ* is the gyromagnetic ratio, Δυ_x_ is the pixel size (mm) in the phase encoding direction and BWx is the pixel BW. The phase images are wrapped between −*π* and +*π* and were unwrapped using an two‐dimensional phase unwrapping algorithm available in FSL.^16^ For EPI‐DWI, the pixel shifts predominantly occur in the phase encoding direction whereas for TSE‐DWI, the shifts occur in the frequency encoding direction. The pixel BW along the phase encoding direction for EPI‐DWI is calculated as

$$BW=\frac{1}{\left(ES*ETL\right)}$$

where ES is the echo spacing and ETL is the echo train length. Please note that the echo spacing and echo train length values obtained were calculated after applying for SENSE, partial Fourier or phase oversampling. The gross tumor volumes (GTVs) drawn on the T2‐w image were overlaid on the field map after registration between T2 and the magnitude image. The mean, standard deviation, minimum, and maximum values of pixel shifts within the GTV ROI were then calculated.

### 2.F Image analysis

SNR comparison was performed between EPI‐ and TSE‐DWI on the b = 0 image. Because the vendor has an algorithm which zeroes background signal outside the body, we placed an ROI in the lowest signal intensity, artifact‐free area in the lung to determine a noise value. Multiple lung/noise areas on multiple slices were averaged and the standard deviation was used as a noise value. For each patient, the two‐dimensional ROIs from EPI‐DWI and TSE‐DWI were exported as DICOM RT structures from the treatment planning system and read into a MATLAB^™^ (MathWorks Inc, MA, USA) program. A pixel‐by‐pixel fit of the ADC, based on monoexponential behavior, was calculated using equation (1) and histograms were generated. From each ADC histogram, the following descriptive parameters were calculated: mean, median, standard deviation, kurtosis, and skewness. These parameters were compared between EPI‐ and TSE‐based DWI for the entire population. The statistical correlation between EPI‐DWI and TSE‐DWI was determined using the paired student's t test. A *P* value less than 0.05 was considered statistically significant.

## 3 RESULTS

### 3.A Phantom measurements

**Figure 2:**
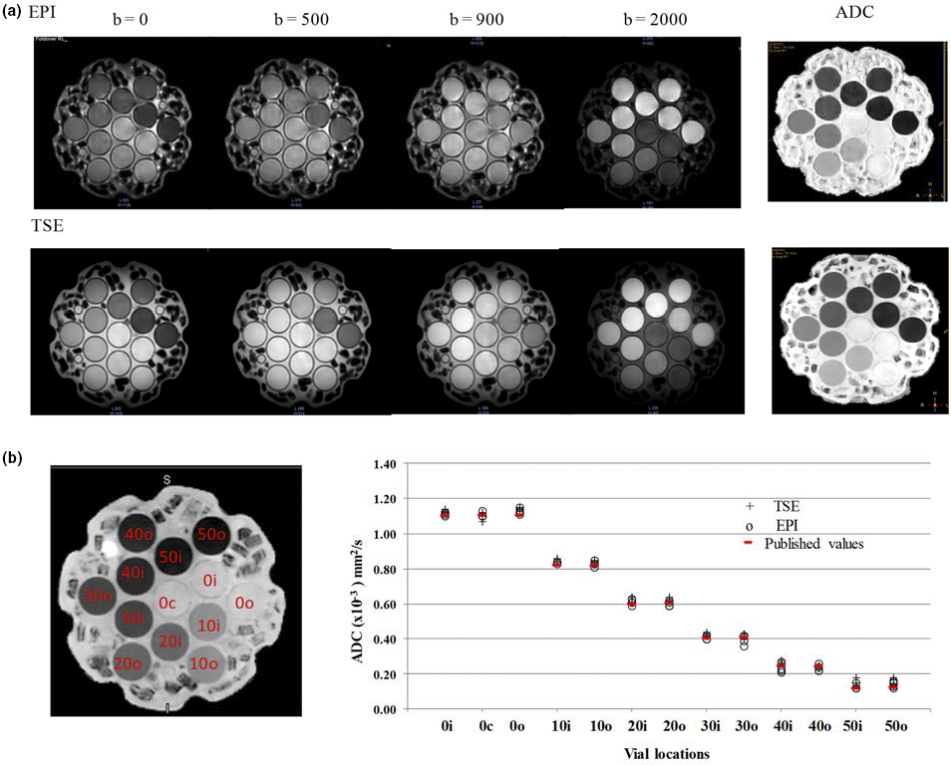
(a) Coronal slice of the diffusion phantom acquired with b values of 0, 500, 900, and 2000 mm^2^/s and apparent diffusion coefficient (ADC) maps derived from the echo‐planar imaging (EPI) and turbo spin‐echo (TSE)‐based acquisitions. (b) The regions of interest drawn in each vial on the ADC map and a plot of ADC vs vial location for TSE‐ and EPI‐based diffusion‐weighted imaging.

Phantom images showed less susceptibility distortion with TSE‐DWI as compared with EPI‐DWI for all b values. This can be observed in Fig. 2 where the circular cross‐sections of the embedded tubes appear distorted in the EPI images. ADC statistics within each ROI were calculated for two same day acquisitions and two different dates (four sets of ADC values for both EPI and TSE acquisitions). These are plotted as a function of vial concentration along with the published values for the phantom in Fig. 2(c). The average ADC difference for all vials between EPI‐ and TSE‐DWI was −0.01 ± 0.008 × 10^−3^ mm^2^/s with ADC TSE systematically higher than EPI, but not statistically significant. The average ADC differences for all vials for EPI‐DWI and TSE‐DWI, with respect to published reference ADC values, were 0.00 ± 0.011 and 0.02 ± 0.012 × 10^−3^ mm^2^/s, respectively. On average, both EPI and TSE values were within 0 ± 3% and 3 ± 2%, respectively, with the published reference values for all the vials, excluding vials 50i and 50o, which showed differences of 14% (0.02 × 10^−3^) and 25% (0.03 × 10^−3^) for TSE‐DWI and 8% (0.009 × 10^−3^) and 13% (0.016 × 10^−3^) for EPI‐DWI, respectively. These two vials also had the lowest ADC values of approximately 0.12 × 10^−3^ mm^2^/s, which are typically below the range of physiologically possible tumor ADC values.

### 3.B Patient study

SNR comparison performed between the EPI‐ and TSE‐DWI image varied between patients as shown in Table 3. SNR value for one patient could not be calculated reliably due to excess noise in the low‐density lung in the EPI image. The comparison showed that both techniques have comparable SNR.

**Table 3:** SNR ratio between EPI and TSE‐based acquisition (SNR_EPI_/SNR_TSE_)

Pat	b = 0 s/mm^2^
1	0.86
2	0.88
3	1.22
4	1.19
5	–
6	1.12
7	1.17
8	0.93
9	0.94
10	0.87
	1.02 ± 0.15

**Figure 3:**
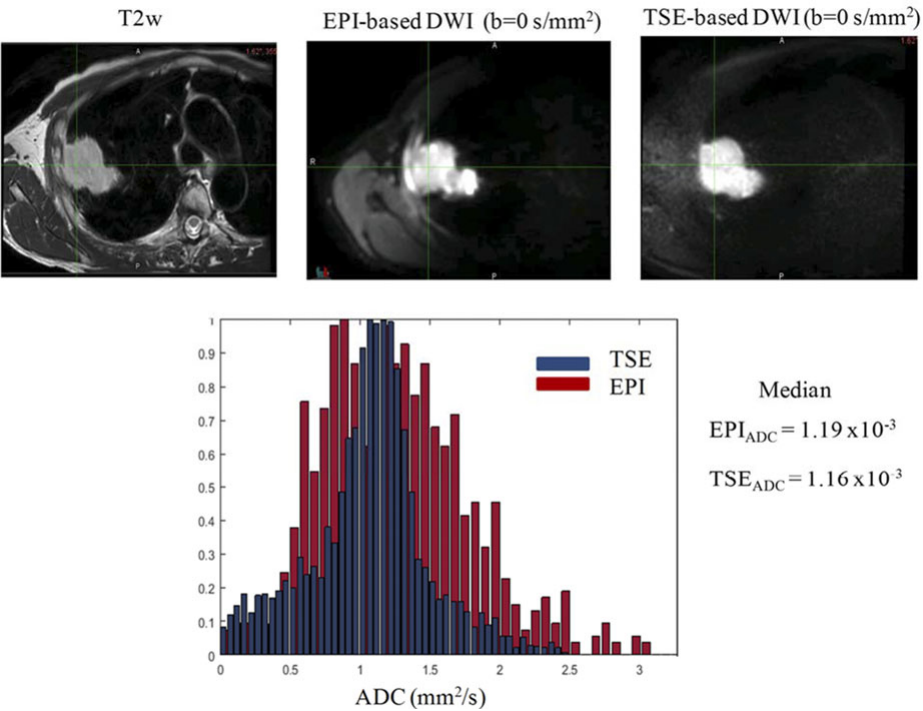
Example of EPI‐ and TSE‐ DWIs along with T2w MRI in patient 2. All images are registered with respect to T2w MRI. Histograms demonstrate the distribution of apparent diffusion coefficient (ADC) values.

Figure 3 shows a comparison of EPI‐ and TSE‐based DW images for another example case along with ADC histograms of the tumor ROIs for both acquisitions. The median ADC for this example case was comparable (1.19 × 10^−3^ vs 1.16 × 10^−3^). The skewness and kurtosis were 0.55, 2.97 for EPI‐ and −1.19 and 4.3 for TSE‐based acquisition. Figure 3 also shows the histograms from EPI‐DWI and TSE‐DWI overlaid on top of each other to show the shape difference for this example case.

**Figure 4:**
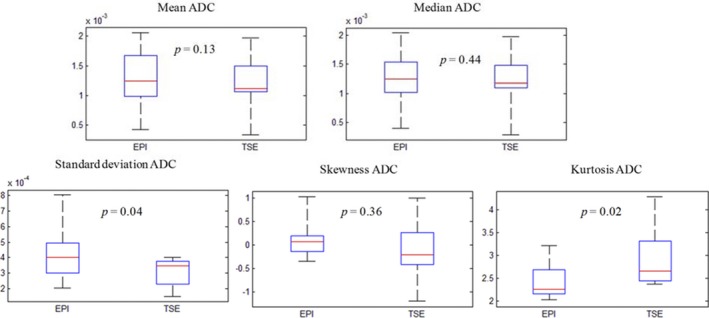
Box plots comparing mean, median, standard deviation, skewness, and kurtosis of EPI‐ and TSE‐based ADC for all the patients along with their statistical significance.

Table 4 shows the mean and standard deviation of ADC values of all 10 patients. The average mean ± SD ADC values for the population were 1.282 ± 0.424 × 10^−3^ mm^2^/s and 1.211 ± 0.311 × 10^−3^ mm^2^/s, respectively, for EPI‐ and TSE‐based acquisitions. The average median, skewness, and kurtosis values for EPI and TSE acquisitions were 1.259 ± 0.458 × 10^−3^, 1.22 ± 0.439 × 10^−3^, 0.138 ± 0.4, ‐0.063 ± 0.699 and 2.43 ± 0.405, 2.89 ± 0.62, respectively. The box plots for the population were obtained for mean, median, standard deviation, skewness, and kurtosis as shown in Fig. 4. Except standard deviation and kurtosis, none of the other variables were statistically significant.

**Table 4:** Mean (and standard deviation) in apparent diffusion coefficient values for echo‐planar imaging (EPI) and turbo spin‐echo (TSE)‐based diffusion‐weighted imaging (DWI)

Patient	EPI (×10^−3^)	TSE (×10^−3^)
1	0.992 (0.302)	1.005 (0.311)
2	1.233 (0.0442)	1.129 (0.202)
3	0.422 (0.203)	0.342 (0.227)
4	1.444 (0.495)	1.187 (0.383)
5	0.811 (0.332)	1.061 (0.329)
6	2.055 (0.585)	1.966 (0.031)
7	1.26 (0.377)	1.109 (0.401)
8	1.157 (0.427)	1.087 (0.363)
9	1.773 (0.804)	1.727 (0.371)
10	1.675 (0.442)	1.498 (0.377)
Average	1.282 (0.424)	1.211 (0.311)

### 3.C Distortion analysis

**Figure 5:**
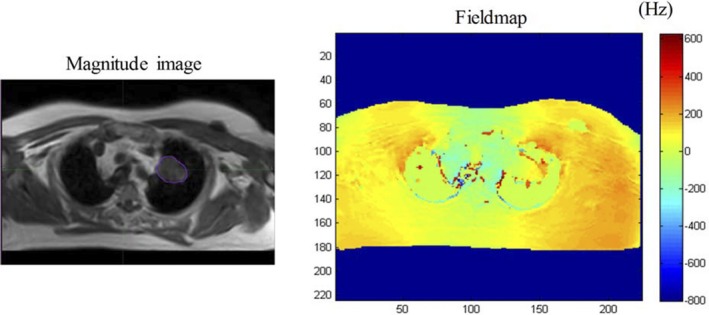
Distortion analysis based on field maps (in Hz) of an example patient. The magnitude image is shown on the left and fieldmap on the right. The magenta contour represents the tumor ROI on the magnitude images.

Distortion analysis showed that the mean shift in the GTVs for the three patients were 13.72 ± 8.12 mm for EPI‐DWI, with a mean average minimum and maximum shift of −24.21 and 36.9 mm, respectively. For TSE‐DWI, the mean, minimum, and maximum shift over both GTVs was 0.61 ± 0.4, −1.08, and 1.65 mm, respectively. Figure 5 shows the field maps in Hz for one example case. As shown in Table 1, the pixel BW of EPI was 34 Hz whereas that of TSE was 757 Hz. For the same pixel size, the extent of distortion in EPI would be 20× that of the TSE acquisitions. One of the patients (#10) had tumor next to heart. The presence of beating heart next to the tumor affected phase unwrapping of this patient resulting in inaccuracies in the phase maps. Without this patient, the mean, minimum, and maximum shift was 2.0 ± 6.2, −34.4, and 26.7 mm for EPI‐DWI and 0.09 ± 0.27, −1.54, and 1.19 mm, respectively for TSE‐DWI.

**Figure 6:**
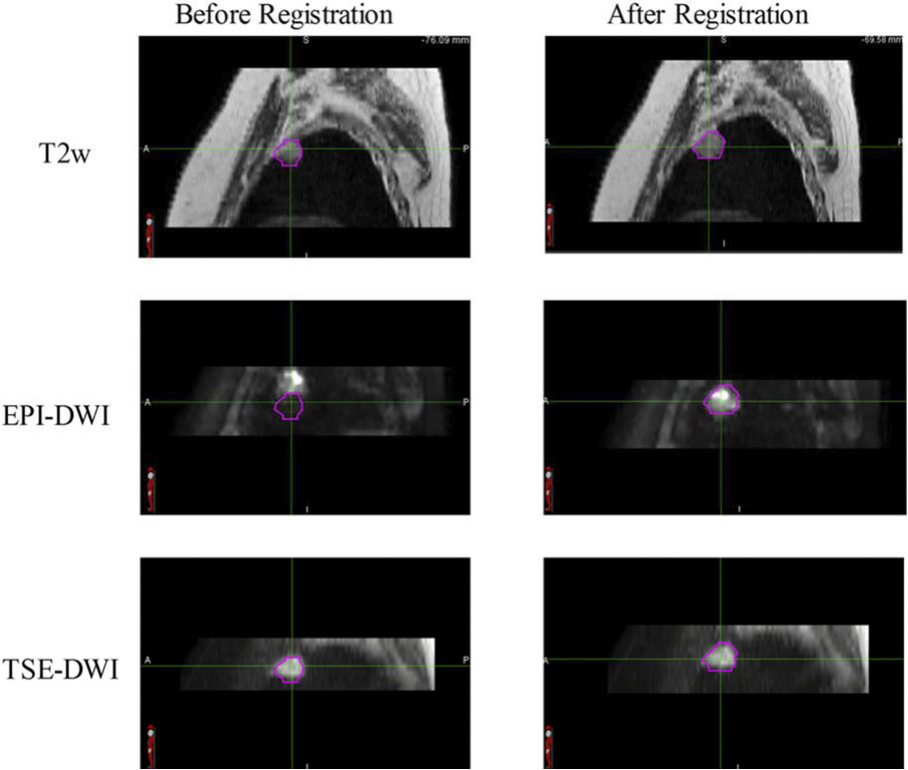
Sagittal orientation of a patient scan representing tumor location before and after manual registration. The figure shows T2w MRI (top row), EPI‐based DWI (middle row) and TSE‐based DWI (bottom row). EPI‐DWI shows a 1.5 cm shift in tumor center location because of susceptibility artifact shown by the location of the tumor contour on the EPI image. The tumor location is corrected after manual registration. The susceptibility artifact on TSE‐DWI is negligible.

The above analysis is also consistent with the amount of shift required to align the tumor location on EPI image with the tumor location on T2w MRI. Figure 6 shows a sagittal view of an example case before and after registration. Before registration, the images are linked with respect to the dicom coordinates. The GTV contour drawn on the T2w MRI is overlaid on the EPI and TSE‐DWI. The susceptibility artifact due to lung‐tumor interface results in >1 cm shift in the tumor center location in the EPI image. Please note that the susceptibility artifact resulting from pixel lumping cannot be completely removed using registration. The shift in tumor location on the TSE image is negligible.

## 4 DISCUSSION

In this study, the tumor ADC histograms derived from an SS‐EPI‐based acquisition and an SS‐TSE‐based acquisition were compared. SS‐TSE‐DWI was superior to EPI in minimizing susceptibility artifacts as shown by the field map analysis. With TSE‐DWI, the geometric accuracy is of the order of a standard anatomic T2w imaging. Minimum distortion allowed easy registration and transfer of contours between T2w and TSE‐DWI. With EPI, there was often a shift in the tumor position and lumping of pixels at the tumor‐air interface due to susceptibility artifacts. This distortion required registration. The low geometric accuracy of EPI‐DWI makes it challenging to incorporate the imaging modality into radiation therapy treatment planning.

Apparent diffusion coefficient values analyzed using a DWI distortion phantom at 0°C showed good accuracy between EPI‐ and TSE‐based acquisitions. In terms of patient studies, the results showed that the mean and median ADC values obtained using EPI‐DWI and TSE‐DWI were not statistically different. In patient 4, the difference between EPI and TSE ADC was substantial, but this may have been due to the proximity of the tumor to the heart. This study did not involve the use of cardiac triggering. Although the mean tumor ADC values were not statistically different, the histograms were more peaked/narrower for TSE‐based distribution as compared with EPI, as shown in Fig. 3 and evident from the different skewness and kurtosis values shown in the box plot in Fig. 4. We believe blurring due to T2 relaxation in the echo train length may have contributed to the long tail in the TSE histograms and needs further investigation. The EPI sequence was respiratory‐triggered but the TSE sequence was acquired free breathing. It was impossible to acquire respiratory‐triggered TSE‐DWI because the scan time would have been prohibitive at approximately 30 min. Studies in the liver have shown that respiratory triggered‐based EPI‐DWI shows similar ADC values compared with free breathing EPI.^17^ While another study found that respiratory‐triggered DWI showed larger variability than breathhold and free breathing.^18^ Although mean and median ADC values did not differ between EPI and TSE, we cannot claim that our study is not influenced by respiratory management technique used between the two techniques. Future study will investigate similar respiratory techniques for both EPI‐ and TSE‐based acquisition and its impact on ADC values. Most studies concerning the role of DWI in lung tumors have used free breathing acquisitions.^1^, ^19^, ^20^, ^21^ Previously reported studies also did not report histogram skewness and kurtosis values. The skewness and kurtosis of the tumor ADC data histogram were calculated to see if the ADC distributions indicate a non‐Gaussian behavior.

With DW‐imaging of the lung, the potential exists for signal variations and increased uncertainty in ADC calculations due to motion. While we did not observe extensive artifacts attributable to motion, it is possible that ADC measurements in lung tumors close to the heart may demonstrate greater uncertainty. Techniques addressing motion in DWI such as rejection of individual frames before averaging 22 may improve data quality.

TSE‐based DWI requires very high TR (>8000 ms) to achieve sufficient SNR for tumor delineation. This results in longer scan times than EPI‐based DWI. The choice between EPI‐DWI and TSE‐DWI is not always clear‐cut. The reduced susceptibility‐induced distortion in TSE‐DWI is a distinct advantage. Multishot TSE brings the potential to use shorter echo trains and increase SNR. When coupled with respiratory triggering to minimize intershot motion artifacts, multishot TSE‐DWI may be the optimal solution for DWI of the lung. For tumors close to the heart, the use of flow compensation and cardiac triggering may further increase the quality of lung TSE imaging at the expense of increased scan time.

One of the weaknesses of this study was that the image acquisitions for EPI and TSE were not of the same duration (3–5 min for EPI and 8 min for TSE) and therefore the SNR per unit time differed. The TSE series scan time was greater due to increased signal averaging in order to enhance SNR. This was necessary because the longer TE and larger inherent BW in the TSE sequence reduced SNR. In preliminary volunteer studies where the number of averages varied, it was determined by our clinicians that an approximate 8‐min scan time was needed to obtain images of usable quality and SNR.

## 5 CONCLUSIONS

In this study, EPI‐based DWI‐ and TSE‐based DWI acquisitions for lung tumors were compared. DWI‐TSE showed much higher geometrical accuracy compared with EPI‐DWI and has the potential for accurate target delineation for radiotherapy applications. Mean and median ADC values were similar with both acquisitions, but the shape of the histograms differed. Future studies will investigate the use of multishot TSE implementation of DWI as well as the effect of respiratory‐triggered TSE acquisition on ADC statistics.

## CONFLICT OF INTEREST

No conflicts of interest.
